# Comparative analysis of the diagnostic performance of five commercial COVID-19 qRT PCR kits used in India

**DOI:** 10.1038/s41598-021-00852-z

**Published:** 2021-11-10

**Authors:** J. Singh, A. K. Yadav, A. Pakhare, P. Kulkarni, L. Lokhande, P. Soni, M. Dadheech, P. Gupta, N. Masarkar, A. K. Maurya, S. Nema, D. Biswas, S. Singh

**Affiliations:** 1 Department of Microbiology, All India Institute of Medical Sciences, Bhopal, 462020 India; 2 Present Address: Translational Medicine Centre, All India Institute of Medical Sciences, Bhopal, India; 3 Department of Biochemistry, All India Institute of Medical Sciences, Bhopal, India; 4 Department of Community & Family Medicine, All India Institute of Medical Sciences, Bhopal, India

**Keywords:** Microbiology, Molecular biology, Diseases, Medical research

## Abstract

To meet the unprecedented requirement of diagnostic testing for SARS-CoV-2, a large number of diagnostic kits were authorized by concerned authorities for diagnostic use within a short period of time during the initial phases of the ongoing pandemic. We undertook this study to evaluate the inter-test agreement and other key operational features of 5 such commercial kits that have been extensively used in India for routine diagnostic testing for COVID-19. The five commercial kits were evaluated, using a panel of positive and negative respiratory samples, considering the kit provided by National Institute of Virology, Indian Council of Medical Research (2019-nCoV Kit) as the reference. The positive panel comprised of individuals who fulfilled the 3 criteria of being clinically symptomatic, having history of contact with diagnosed cases and testing positive in the reference kit. The negative panel included both healthy and disease controls, the latter being drawn from individuals diagnosed with other respiratory viral infections. The same protocol of sample collection, same RNA extraction kit and same RT-PCR instrument were used for all the kits. Clinical samples were collected from a panel of 92 cases and 60 control patients, who fulfilled our inclusion criteria. The control group included equal number of healthy individuals and patients infected with other respiratory viruses (n = 30, in each group). We observed varying sensitivity and specificity among the evaluated kits, with LabGun COVID-19 RT-PCR kit showing the highest sensitivity and specificity (94% and 100% respectively), followed by TaqPath COVID-19 Combo and Allplex 2019-nCoV assays. The extent of inter-test agreement was not associated with viral loads of the samples. Poor correlation was observed between Ct values of the same genes amplified using different kits. Our findings reveal the presence of wide heterogeneity and sub-optimal inter-test agreement in the diagnostic performance of the evaluated kits and hint at the need of adopting stringent standards for fulfilling the quality assurance requirements of the COVID-19 diagnostic process.

## Introduction

The ongoing COVID-19 pandemic has spread globally infecting approximately 90 million individuals and has led to 1.9 million deaths within a year since the first declaration of the outbreak in Wuhan, China on 31st December 2019^1,2^. Since the overwhelming majority of the infected cases are asymptomatic^3,4^ widespread testing has been advocated to be critical for the early diagnosis of cases and containment of the pandemic.

The availability of whole genome sequencing of the SARS-CoV-2 virus, within 2 weeks of the declaration of the outbreak, enabled the development of a wide range of RT-qPCR kits by several commercial manufacturers within a relatively short span of time. To cope with this urgency and huge demand for testing, regulatory agencies like United States Food and Drug Administration (USFDA) adopted the procedure of Emergency Use Authorization (EUA) for clinical use of these kits in contrast to the customary process of granting full approval or clearance for diagnostic assays^5,6^.

Consistent with this worldwide trend of rapid ramp-up of diagnostic capacity, in India over a period of nine months 1260 diagnostic laboratories have been approved by the Indian Council of Medical Research (ICMR), which is spearheading the country’s response to the pandemic. To perform the diagnostic RT-qPCR tests, ICMR has approved 145 commercial kits till date^7,8^. These kits target a wide range of viral genes, including RNA-dependent RNA polymerase (RdRp), open reading frame (ORF1ab), envelope (E), nucleocapsid (N), and spike (S) proteins^9^. However, there have been limited studies that have compared these kits relative performances via inter-test agreement of these kits using a robust panel of clinical samples within the operational settings of a diagnostic virology laboratory^9–14^.

Since this exercise constitutes a critical part of the total quality management of a diagnostic set-up, we undertook this study to evaluate the analytical and clinical performance of 5 commercial SARS-CoV-2 molecular diagnostic assays that have been granted EUA by the FDA. These kits included Allplex 2019–nCOV assay, Lab Gun COVID-19 RT-PCR Kit, TaqPath COVID-19 combo kit, BGI Real Time Fluorescent RT-PCR Kit and TRUPCR SARS-CoV-2 RT qPCR KIT assays and their performance was compared with 2019-nCoV Kit, provided by National Institute of Virology (NIV) under the aegis of ICMR.

## Materials and methods

Since the study was done on anonymized samples after they have been used for routine diagnostic processing; involved less than minimal risk to the participants as there was no additional sampling involved; was integrated with the quality audit of the institute and the programmatic requirement of evaluating the operational performance of the commercial diagnostic kits, it qualified for exemption from ethical review and waiver of informed consent according to ICMR’s “National Ethical Guidelines for Biomedical and Health Research involving Human Participants”.

We created a panel of positive samples from individuals having suggestive clinical features, identifiable history of contact with known cases of COVID-19 and positive laboratory report in ICMR-approved 2019-nCoV Kit, provided by National Institute of Virology, Pune. Similarly, a panel of healthy control samples was created from asymptomatic individuals without any known contact with cases of COVID-19. Furthermore, a panel of disease controls was created from individuals diagnosed with five other respiratory viruses like Influenza A H1N1 pdm09, Influenza A H3N2, Influenza B and Respiratory Syncytial virus, Human Metapneumo virus.

Nasopharyngeal and oropharyngeal swab samples were collected from individuals belonging to each of the three groups mentioned above. Sterile nylon, dacron or rayon swabs with flexible plastic shafts were used to collect nasopharyngeal specimens (NPS) from symptomatic patients. After collection, swabs were placed in sterile Virus Transport Medium (VTM). Before testing, samples were vortexed for 3–5 s and a calibrated pipette was used to transfer the specimen volume specified by the kit manufacturer for RNA extraction. To simulate actual testing conditions in a COVID-19 diagnostic laboratory, the samples were not tested in replicates.

RNA extraction from the collected samples was done using HiPurAViral RNA purification kit manufactured by HiMedia Laboratories Pvt. Ltd., India as per the kit instructions, after removing all patient identifiers. Briefly, 200 µl of patient sample was added to 460 µl of lysis buffer mixture. It was mixed well and incubated at room temperature for 10 min. The magnetic beads in the buffer were separated from the suspension using the magnetic stand, followed by washing with buffer MW1, MW2 and with absolute ethanol. RNA was eluted by incubating the beads with RNase free water at 56 $^\circ$C. The eluted RNA was stored at −80 $^\circ$C till further use.

The volume of extracted RNA sample, specified by the respective kit manufacturers, was subjected to diagnosis using 2019-nCoV Kit (ICMR-NIV Pune), Allplex 2019–nCOV assay, Lab Gun COVID-19 RT PCR Kit, TaqPath COVID-19 combo kit, BGI Kit Real Time Fluorescent RT-PCR Kit and TRUPCR SARS-CoV-2 RT qPCR KIT assays for detecting SARS-CoV-2 as per the manufacturer’s protocol on a ABI7500 thermal cycler (Applied Biosystem).

The target genes, performance characteristics and operational aspects of the evaluated genes are depicted in Supplementary table-1, based on the information provided in the respective kit inserts. The PCR mix and thermal profiles were also used according to the kit protocols (Supplementary tables 2 & 3).

As recommended by the manufacturers, a sigmoidal curve with a specific Ct value was considered as the criterion for considering a sample as positive for SARS-CoV-2. The criteria provided by the manufacturers of the respective kits were used for interpretation of the results (Supplementary table 1). All the required controls namely no-template control, extraction control and positive control were tested simultaneously with every set of samples, as part of the quality control of the procedure. The data was entered in MS Excel. The descriptive summarization of variables of interest was done by median and IQR for ordinal and interval data and counts for nominal data. Sensitivity and specificity were determined, as per convention, using confirmed COVID-19 cases and healthy and diseased control panels. To check the proportion of agreement, Cohen’s kappa test was used. Lin’s Concordance correlation coefficient of Ct values was also estimated and Bland Altman plots were used for understanding agreement in Ct values among different methods.

All the analyses were done by using base R version 4.0.3, tidyverse, epi-R^15–18^ and 'Bland Altman Leh'packages^19^.

## Results

In absence of a gold standard assay, we considered a combination of clinical, epidemiological and laboratory criteria as indicator of COVID-19 infection. Accordingly, a panel of 92 clinical samples were collected from equal number of cases who were symptomatic for respiratory infection, had history of contact with diagnosed cases of COVID-19 and were RT-qPCR positive by 2019-nCoV kit provided by National Institute of Virology, under the aegis of Indian Council of Medical Research (ICMR), which is the apex national agency for COVID-19 testing. Similarly, a panel of 60 control samples was created, including healthy individuals and patients infected with other respiratory viruses (n = 30, in each group).

We observed varying sensitivity and specificity among the evaluated kits, with LabGun COVID-19 RT-PCR kit showing the highest sensitivity and specificity (94% and 100% respectively), followed by TaqPath COVID-19 Combo and Allplex 2019-nCoV assay (Table 1).

**Table 1:** Obtained sensitivity and specificity of various RT-qPCR Kits in this study on COVID-19 positive cases.

S.no	Name of the Kit	Sensitivity % (95% CI)	Specificity (%) (95% CI)	Kappa coefficient (95% CI)
1	TaqPath COVID-19 Combo Kit	88 (79.85–93.19)	100 (93.98–100)	0.8532 (0.696–1.01)
2	LabGun COVID-19RT-PCR Kit	94 (86.49–96.98)	100 (93.98–100)	0.918 (0.76–1.077)
3	Allplex 2019-nCoV Assay	88 (79.85–93.19)	100 (93.98–100)	0.8532 (0.696–1.01)
4	BGI Kit Real-Time Fluorescent RT-PCR Kit for Detecting SARS-CoV-2	80 (71.18–87.25)	100 (93.98–100)	0.764 (0.61–0.919)
5	TRUPCR Kit	73 (62.96–80.86)	100 (93.98–100)	0.679 (0.528–0.829)

We estimated inter-test agreement between the NIV kit and the five other evaluated kits. Of the panel of 92 positive samples, 52 tested positive by all the kits. Furthermore, an additional set of 34, 28, 29, 22 and 15 samples tested positive by LabGun, TaqPath, Allplex, BGI and TRUPCR kits respectively. The best inter-test agreement with NIV kit was shown by LabGun. Considering kappa value of 0.8 to be an indicator of ‘strong’ inter-rater agreement^20^, we observed that two of the five evaluated kits demonstrated sub-optimal performance in comparison to the NIV kit. The performance of none of the five kits could be considered to be significantly superior relative to others, owing to overlapping confidence intervals. (Table-1). The comparative distribution of Ct values, as observed on testing with the evaluated kits, is depicted in Table 2.

**Table 2:** Ct values estimated by different kits using mean and SD as well as median and interquartile range.

Characteristic	NIV	Taq path	Lab Gun	Allplex	BGI	TRUPCR
**E-Gene**						
N	92.0	0.0	89.0	80.0	0.0	81.0
Mean (SD)	28.2 (4.4)		28.1 (6.2)	27.1 (6.0)		26.5 (5.7)
Median (IQR)	27.8 (24.9, 31.4)		27.7 (23.0, 32.4)	26.2 (22.6, 31.4)		25.4 (22.2, 31.0)
**RdRp**						
N	92.0	0.0	86.0	76.0	0.0	68.0
Mean (SD)	30.9 (5.1)		27.8 (6.1)	28.5 (5.6)		26.5 (4.5)
Median (IQR)	31.7 (26.9, 34.7)		27.1 (22.5, 32.5)	27.4 (24.4, 32.9)		26.0 (23.4, 29.0)
**ORF**						
N	92.0	78.0	0.0	0.0	77.0	0.0
Mean (SD)	28.0 (4.8)	24.0 (4.4)			27.2 (5.9)	
Median (IQR)	28.7 (24.6, 32.0)	24.1 (21.0, 28.0)			26.0 (22.7, 30.7)	
**N-gene**						
N	0.0	79.0	0.0	83.0	0.0	0.0
Mean (SD)		24.8 (5.6)		26.9 (5.8)		
Median (IQR)		24.2 (19.9, 29.4)		25.8 (22.3, 31.6)		
**S-Gene**						
N	0.0	78.0	0.0	0.0	0.0	0.0
Mean (SD)		24.3 (5.2)				
Median (IQR)		23.5 (20.4, 27.7)				

Interestingly, the degree of agreement was not dependent on the number of gene targets that were similar between the kits. To illustrate, TaqPath and Allplex kits showed identical kappa values though they had 1 and 2 gene targets in common with NIV kit respectively. Inter-test agreement was also compared between samples with low and high viral loads by restricting analysis to samples showing Ct values ≤ 35 (n = 28) and ≤ 30 (n = 64) in the reference kit. We did not observe improved concordance between the kits, in samples with relatively higher viral load.

**Figure 1:**
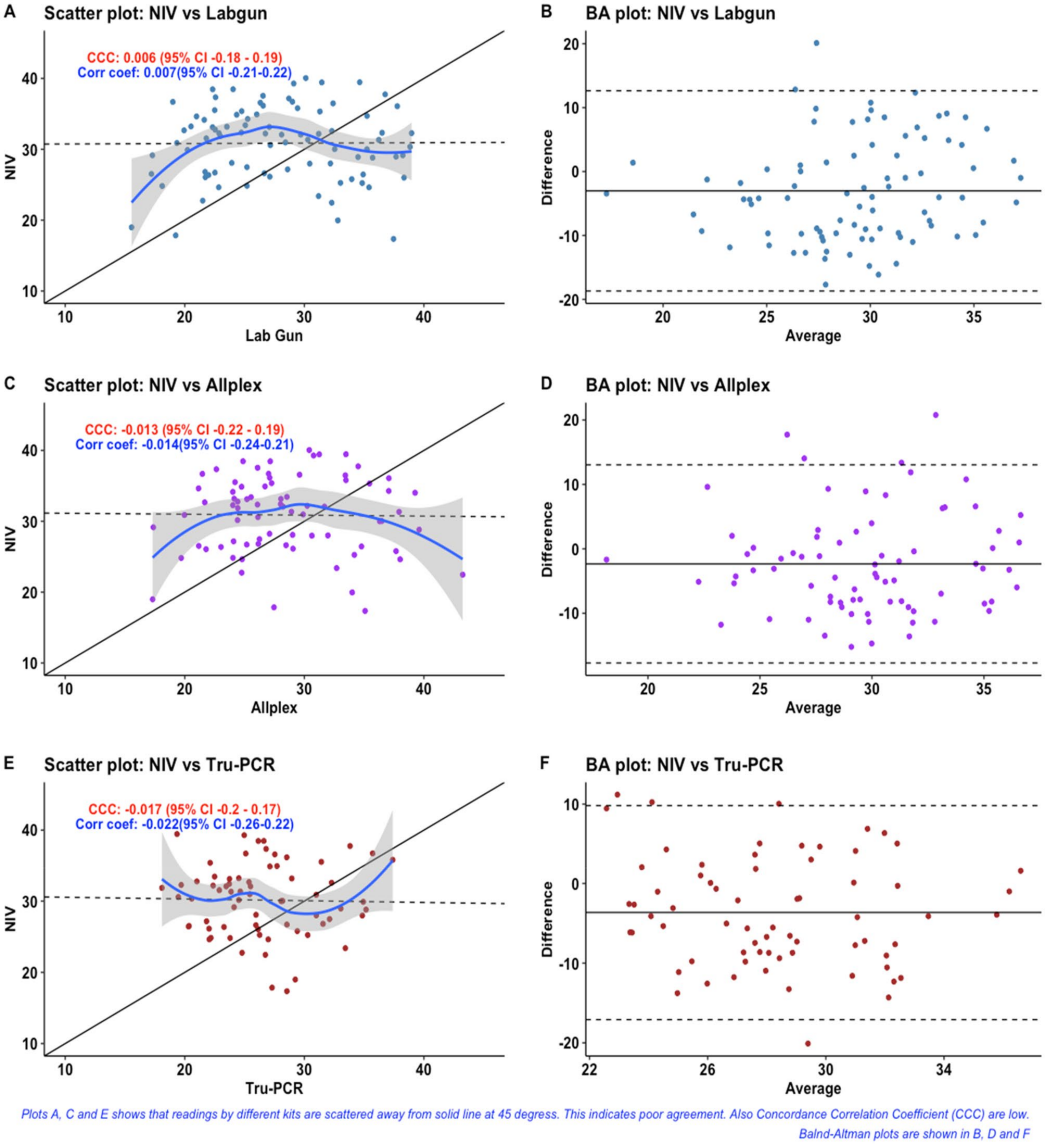
Scatter and Bland -Altman plots for RdRp-Ct values of different kits.

**Figure 2:**
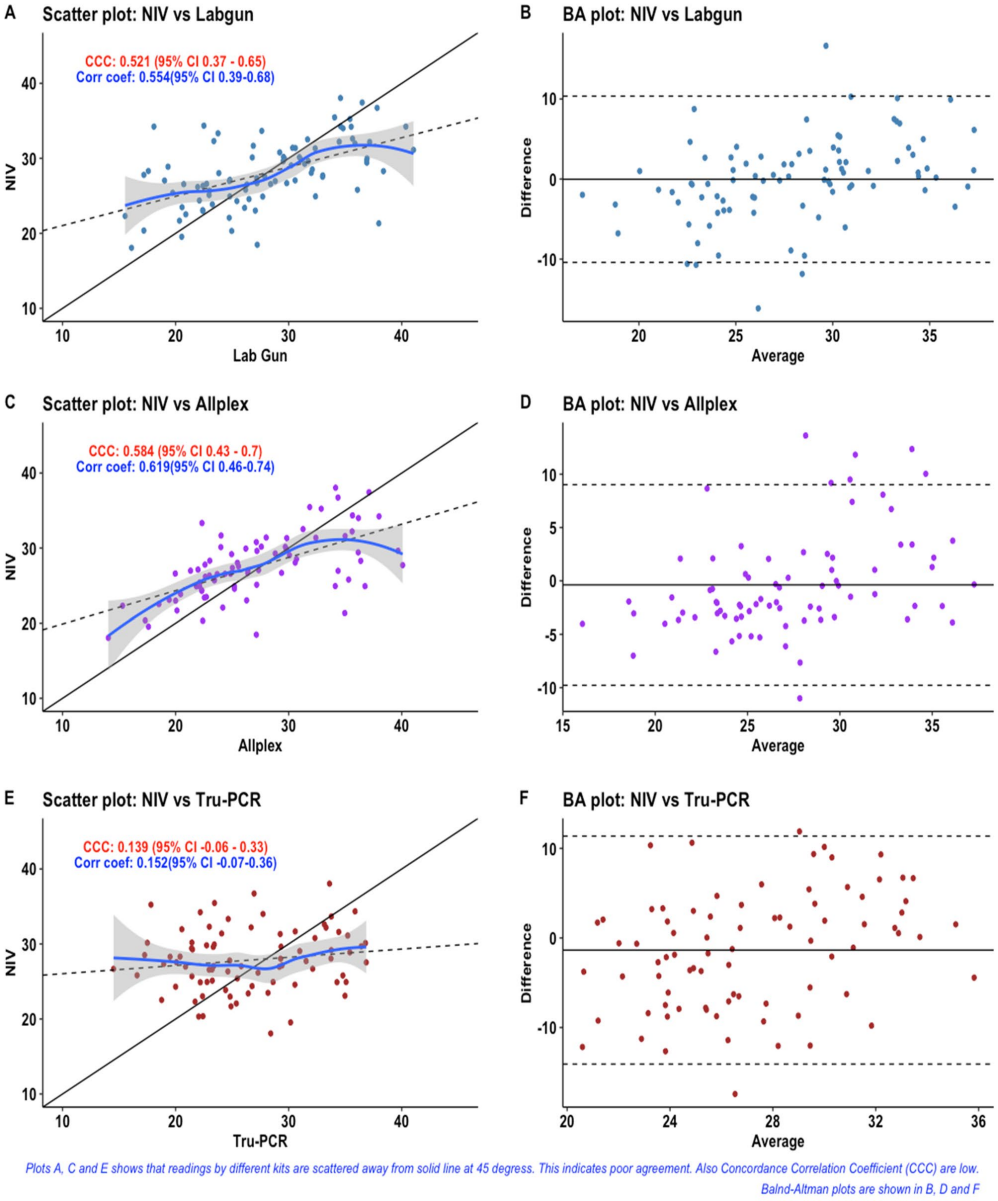
Scatter and Bland -Altman plots for E gene-Ct values of different kits.

**Figure 3:**
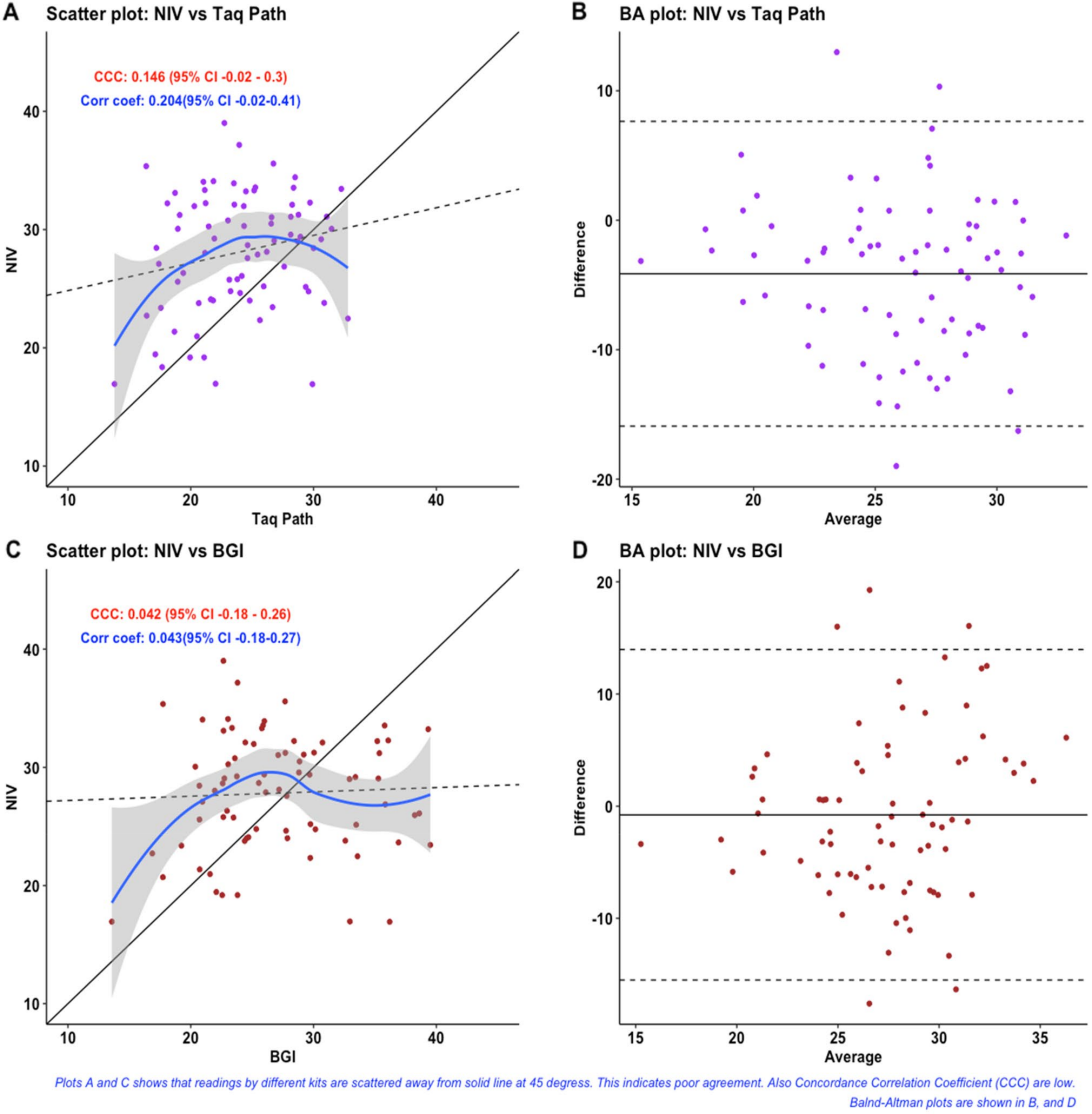
Scatter and Bland -Altman plots for ORF1ab gene-Ct values of different kits.

We next examined the correlation of Ct values observed on testing the panel of samples with kits having same gene targets. We used Lin’s Concordance Correlation Coefficient (CCC) for this analysis^21^. Lin’s Concordance Correlation Coefficient (CCC) is used to examine agreement between two continuous measurements. Like Pearson’s correlation coefficient, CCC ranges from − 1 to + 1. However, if one method of measurement differs systematically from other method then we will get higher value of correlation coefficient but concordance is not ensured. Therefore, Lin’s CCC is better measurement when our aim is to assess agreement or concordance. Lin’s CCC was estimated between the evaluated kits and the reference kit for E, RdRP and ORF1ab genes (Figs. 1, 2 and 3). Lin’s CCC for Ct values observed on amplifying the E gene with Tru-PCR and NIV kits was not statistically significant. On the other hand, the correlation coefficients for both RdRp and ORF-1ab genes were not significant for any of the evaluated kits.

We also evaluated the kits for several operational features including turn-around time, instrument compatibility, reagent cost and input volume of RNA. All the five kits except TaqPath COVID-19 Combo kit were compatible with ABI7500, Bio-Rad CFX96 and Cobas z 480 ROCHE real time PCR machine. TaqPath was only compatible with ABI7500 machine. PCR duration was maximum for LabGun COVID-19 RT-PCR kit and minimum for BGI kit (Supplementary table 2).

When we create a scatter plot showing measurement by one method on X-axis and by other on Y-axis, then their values should be clustered near 45-degree diagonal line if there is high agreement. Solid line indicates this 45-degree line. Dashed line is fit line for linear regression analysis.

## Discussion

In this study we report a comparative assessment of five commercially available RT-qPCR kits that have been recently validated for COVID-19 diagnostics in India. Using a panel of anonymised samples, curated on the basis of clinical, epidemiological and laboratory criteria, we observed significant heterogeneity in the clinical performance and operational efficiency of the different commercial kits compared to the reference kit provided by the national agency of India. The wide variability in performance spanned across the range of viral load in the clinical samples and was not dependent on the number of gene targets that were shared between the evaluated kits and the reference kit. While LabGun COVID-19 RT PCR kit showed the highest sensitivity, specificity and inter-test agreement with the comparator kit, two of the five kits demonstrated sub-optimal concordance with the reference kit (kappa < 0.8). There was poor correlation between the Ct values obtained from the same sample and the same gene target in different kits.

Lack of concordance between different commercial kits tends to be a major cause of irreproducible test results in clinical practice. This implies the need for enforcement of comprehensive quality management practices for kit manufacturers. Availability of a panel of reference standards, containing pre-determined copy numbers of various target genes, could help in resolving such heterogeneity and improving performance standards of the marketed kits. In absence of quality-assured kits, the purpose of containing the pandemic by widespread diagnostic testing cannot be fully achieved.

Though RT-qPCR constitutes the cornerstone in the diagnosis of COVID-19, no kit has been declared to be the gold standard by any regulatory agency till date. The usual samples of nasopharyngeal and oropharyngeal swabs have been reported to deliver sensitivity of only 70% in COVID-19^22^. While our selection of cases were based on the WHO definition of a “confirmed” case of COVID-19 ^23^, our selection of controls enabled us to evaluate the specificity of these kits in discriminating between COVID-19 and other simulating clinical conditions. Evaluating the relative performance of 5 most common diagnostic kits, against a robust panel of clinically and RT-qPCR confirmed cases and other healthy and disease controls, is one of the main strengths of this study.

Our study on delineating the clinical sensitivity and specificity of the evaluated kits assumes significance in view of the fact that the grant of EUA is irrespective of the determination of these performance characteristics. The data considered for EUA comprises of analytical sensitivity (i.e., detection limit) and analytical specificity (i.e., reactivity with potentially cross-reacting organisms). Owing to the novelty of the pathogen and the lack of both reference methods and a clearly defined disease state, establishment of clinical sensitivity and specificity are not required under EUA ^24^.

On comparing the relative diagnostic performance of the evaluated kits, we observed the highest diagnostic accuracy with LabGun COVID-19 RT-PCR kit, in terms of clinical sensitivity and specificity. Strong agreement with the reference kit was observed in case of three of the evaluated kits for dichotomous outcomes, i.e. positive and negative results. In addition to this categorical agreement, we also evaluated the extent of correlation between Ct values obtained on amplifying the same target gene using different commercial kits. In agreement with a previous study, we too observed poor correlation between the Ct values obtained on using the different kits^25^. However, despite the use of Ct values in comparing viral loads between samples collected and tested under similar conditions, the clinical diagnosis and management of COVID-19 is based on the qualitative results of RT-qPCR assays. Hence, categorical agreement can be considered as the yardstick of kit comparability and lack of such agreement between some of the approved commercial kits is worrisome.

The inter-test agreement was not associated with the number of gene targets that were similar between the kits or with the Ct value of the samples. Poor correlation between Ct values of PCR assays targeting the same gene hints at the variable efficiency of primers, probes and master mix components included in the different kits. The variation in efficiency of PCR amplification was found to be irrespective of the viral load, since the inconsistency in inter-test agreement was similar between samples with Ct values above and below 30 or 35.

Though diagnostic performance of COVID-19 RT-qPCR assays have been compared in several earlier studies^2^, the operational aspects of such comparison have not been reported till date. Since the number of RT-qPCR instruments in a particular lab is usually limited, the adaptability of the diagnostic kits to a wider range of instruments is a useful advantage. The LabGun COVID-19 RT -PCR kit performed the best in terms of versatility. Similarly, the turn-around time of the assay becomes important in view of the increasing challenges of space limitation in holding areas for COVID-19 suspects. Though the assay time for LabGun COVID-19 RT -PCR kit was the longest (140 min) among the evaluated kits, still the higher agreement and versatility of this kit over-shadows this apparent disadvantage.

Our study suffered from several limitations. Firstly, the limit of detection of the different kits could not be determined in absence of a suitable RNA standard. Secondly, we could not assess the cross-reactivity of these kits with commonly circulating coronaviruses due to want of samples known to be positive for these viruses. Thirdly, the kit evaluation was done following the thawing of archived frozen samples which may have impacted the performance of the kits. Lastly, though we attempted to select the most widely used kits, we understand that this is a relatively minor proportion of the total number of kits that are approved for diagnostic use in India.

To conclude, the present study advocates stringent verification of a kit’s diagnostic performance and due consideration of its operational aspects before adopting it for clinical use and also highlights the limited inter-test agreement between assays irrespective of the target genes amplified and the viral load present in the samples.

## Supplementary Information

Supplementary Information.
