# Supplementary material for: Comparative analysis of the diagnostic performance of five commercial COVID-19 qRT PCR kits used in India
Source: Sci Rep. 2021 Nov 10;11:22013. doi: 10.1038/s41598-021-00852-z (PMC8580981; doi:10.1038/s41598-021-00852-z)
Supplement: Supplementary file 1 — Supplementary Information. [file 41598_2021_852_MOESM1_ESM.docx]

.**Supplementary table -1: Claimed sensitivity and specificity of different kits for molecular detection of COVID-19**

| **S. No** | **Name of Kit** | **Name of Manufacturer** | **Compatible instruments** | **PCR duration (without Ramp rate)** | **Gene Targets** | **Limit of Detection** | **Claimed Sensitivity** | **Claimed Specificity** | **Homology** | **Threshold Cycle Values (Ct) (for all gene targets)** | Criterion for positive reaction* |
| --- | --- | --- | --- | --- | --- | --- | --- | --- | --- | --- | --- |
| **1** | **2019-nCoV Kit** | ICMR-NIV, Pune | ABI7500, Biorad CFX96, Roche Cobas Z 480 | 70 mins | E, RdRP & ORF1b | - | - | - |  | 35 | Ct value for any 2 viral genes should be < 35. |
| **2** | **LabGun COVID-19RT-PCR Kit** | LabGenomics | ABI7500, BioradCFX96, Roche Cobas Z 480 | 101.25 mins | E & RdRP gene | 20 copies/µl | 95% | 95% | 100% | 40 | Ct value for both viral genes should be < 40. |
| **3** | **Allplex 2019-nCoV Assay** | Seegene | ABI7500, Biorad CFX96, Roche Cobas Z 480 | 69 mins | E, RdRP and N gene | - | - | - | 100% | 35 | Ct value for any 2 viral genes should be < 35. |
| **4** | **TaqPath COVID-19 Combo Kit** | ThermoFisher  Scientific | ABI7500 | 36 mins | ORF1ab, N and S gene | 10 copies/reaction |  | 95% | 100% | 40 | Ct value for any 2 viral genes should be < 40. |
| **5** | **Real-Time Fluorescent RT-PCR Kit for Detecting SARS-CoV-2**  **BGI Kit** | BGI Genomics Co. Ltd | ABI7500, Biorad CFX96, Roche Cobas Z 480 | 25 mins | ORF1ab | 100 copies/mL | High | High | 100% | 37 | Ct value for any 2 viral genes should be < 37 |
| **6** | **TRUPCR SARS-CoV-2 RT qPCR kit** | 3B Black biotech Pvt Ltd | ABI7500, Biorad CFX96, Roche Cobas Z 480 | 57 mins | E & RdRP | 5.2 copies/ reaction | High | High | 100% | 35 | Ct value for any 2 viral genes should be < 35. |

**Supplementary table -2: Reagents for PCR Mix of different kits for rapid detection of COVID-19 (Volume for one reaction)**

| **Reagents** | **2019-nCoV Kit**  (µl) | **LabGun COVID-19RT-PCR Kit**  (µl) | **Allplex 2019-nCoV Assay**  (µl) | **TaqPath COVID-19 Combo Kit**  (µl) | **Real-Time Fluorescent RT-PCR Kit for Detecting SARS-CoV-2**  **BGI Kit** (µl) | **Tru PCR Kit Version one**  (µl) |
| --- | --- | --- | --- | --- | --- | --- |
| Reaction Buffer | 12.5 | 10 | 5 | - | - | 10 |
| Reaction Mix | - | - | - | 6.25 | 18.5 | - |
| Internal Control | - | 1 | Added during RNA extraction | Added during RNA extraction | - | - |
| Enzyme | 1 | 1 | 2 | - | 1.5 | 0.35 |
| Primer probe mix | 1.5 | 4 | 5 | 1.25 | - | 4.65 |
| RNase free water | 5 | - | 5 | 7.5 | - | 5 |
| RNA | 5 | 4 | 8 | 10 | 10 | 5 |
| **Total Volume** | **25** | **20** | **25** | **25** | **30** | **25** |

**Supplementary table -3: Temperature profile for PCR of different kits for rapid detection of COVID-19**

| **Steps** | **2019-nCoV Kit** | | | **LabGun COVID-19RT-PCR Kit** | | | **Allplex 2019-nCoV Assay** | | | **TaqPath COVID-19 Combo Kit** | | | **Real-Time Fluorescent RT-PCR Kit for Detecting SARS-CoV-2**  **BGI Kit** | | | **Tru PCR Kit Version 1** | | | | |
| --- | --- | --- | --- | --- | --- | --- | --- | --- | --- | --- | --- | --- | --- | --- | --- | --- | --- | --- | --- | --- |
| **Initial Hold** |  | Temp. | Time |  | Temp. | Time |  | Temp. | Time |  | Temp. | Time |  | Temp. | Time |  | Time |  | Temp. | Time |
|  | 1X | 50^0^C | 20 m | 1X | 50^0^C | 30 m | 1X | 50^0^C | 20 m | 1X | 25^0^C | 2 m | 1X | 50^0^C | 20 m | 1X | 30 m | 1X | 50^0^C | 15 m |
|  |  | 95^0^C | 10 m |  | 95^0^C | 15 m |  | 95^0^C | 15 m |  | 53^0^C | 10 m |  | 95^0^C | 10 m |  | 3 m |  | 95^0^C | 5 m |
|  |  | | |  | | |  | | |  | 95^0^C | 2 m |  | | |  | | | | |
| **Cycling** | 40X | 95^0^C | 15 s | 45X | 95^0^C | 15 s | 45X | 95^0^C | 15 s | 40X | 95^0^C | 3 s | 40X | 95^0^C | 15 s | 45X | 15 s | 37X | 95^0^C | 5 s |
|  |  | 60^0^C | 30 s |  | 60^0^C | 1m |  | 58^0^C | 30 s |  | 60^0^C | 30 s |  | 60^0^C | 30 s |  | 30 s |  | 60^0^C | 40 s |
|  |  | | |  | | |  | | |  | | |  |  |  |  | | | 72^0^C | 15 s |
